# Accuracy of Emergency Room Triage Using Emergency Severity Index (ESI): Independent Predictor of Under and Over Triage

**DOI:** 10.7759/cureus.20229

**Published:** 2021-12-07

**Authors:** Khalifa Rashid, Maaz Ullah, Syed T Ahmed, Muhammad Z Sajid, Muhammad A Hayat, Bakht Nawaz, Kiran Abbas

**Affiliations:** 1 Department of Emergency Medicine, Hayatabad Medical complex (HMC), Peshawar, PAK; 2 Department of Medicine, Ayub Teaching Hospital, Abbottabad, PAK; 3 Department of Emergency Medicine, MAP Medical Centre, Peshawar, PAK; 4 Department of Emergency Medicine, MAP Medical Center, Peshawar, PAK; 5 Department of Pharmacology, Peshawar Medical College, Peshawar, PAK; 6 Department of Surgery, Ayub Teaching Hospital, Abbottabad, PAK; 7 Department of Medicine, Jinnah Postgraduate Medical Centre, Karachi, PAK

**Keywords:** emergency room, hypoxia, oxygen saturation, triage, bradycardia, tachycardia

## Abstract

Introduction

Patient saturation in emergency care departments is a significant issue that impacts the healthcare system globally. This study was purposed to evaluate the accuracy of the ER triage using the Emergency Severity Index (ESI).

Methodology

A prospective observational study was performed at Hayatabad Medical Complex, Peshawar, from October 2020 to March 2021. Data from one of the second largest hospitals in Khyber Pakhtunkhwa were acquired to carry out this study. All data from our emergency department have been retrieved and recorded using appropriate procedures and software. Triage accuracy has been established by comparing proposed resource consumption (acuity level 3-5) to the actual resources utilized in these hospitals as the amount of an agreement between standard guidelines and local observations. In terms of resource expenditure, we also assessed the interconnection between acuity level and extent of accuracy. SPSS version 21 (IBM Inc., Armonk, New York) was used to document and analyze all of the data.

Results

The greatest odds of undertriage to moderate acuity were associated with age ≥65 years; OR 1.49, 95% CI (1.25-1.72) and OR 2.18 CI (1.22-3.73) for under-triage to low acuity designations. Severe hypoxia, severe bradycardia, and severe tachycardia were all strongly linked with the risk of under-triage of moderate-acuity levels OR 2.19 95% CI (1.49-3.13); OR 2.54 (1.53-4.01); and OR 2.17 (1.61-2.88), respectively. Essentially, there were also significant associations with under-triage to moderate acuity due to the lack of oxygen saturation measurement. Hypertension (≥200mmHg) was linked with increased odds of undertriage to moderate acuity with OR 1.29 95% CI (0.68-2.01). There were no anomalous vital signs associated with an increased likelihood of over-triage to high and moderate ESI acuity levels.

Conclusion

Our study indicated that increasing the age of patients was a significant factor associated with odds of under-triage. Furthermore, certain vital signs, including severe bradycardia, tachycardia, and severe hypoxia, were connected to the risk of under-triage of moderate acuity. Further, large-scale and multicenter studies should be conducted to assess other triage systems, which may provide a more accurate and reliable approach to evaluate the severity of patients’ injuries by the hospital staff and physicians in the emergency room. They should be translated to local languages to assign treatment priorities in a structured and dependable manner.

## Introduction

Patient saturation in emergency care departments is a significant issue that impacts the healthcare system globally. This problem is associated with an increase in demand for healthcare, fewer hospital beds, and a lack of healthcare providers [1]. To allow the efficient running of an emergency system, protocols are introduced which can help stratify the levels of risk that would help accurately assess the patient's clinical status. Triage of patients is essential when service delivery is limited, and requirement exceeds capacity, which can put patients' safety and the quality of care they receive in jeopardy [2].

Under-triage is the inability to classify patients with severe illnesses from those with less urgent needs, which can potentially withhold necessary interventions from those in dire need resulting in significant mortality [3]. On the other hand, over-triage causes allotment of resources to those with non-urgent healthcare needs, resulting in the diversion of limited time and resources from those who desperately require it [4]. The level of triage helps in the allocation of adequate resources to those in need [5].

The Emergency Severity Index (ESI) is currently one of the most widely implemented protocols in the emergency department for efficacious patient triage. This scale stratifies patients according to the severity of their illness by a rough estimation of the level of resources that they require to cater to their ailments. Patients who are categorized as level one require urgent care, while those as level two or three can be treated within a 15-minute window. Those with not-so-urgent needs belong to level four or five and can be addressed within 30 minutes [6].

Enhancing protocols for the classification of risks is a principal goal of emergency services as it improves clinical outcomes and services with limited resources at hand [7]. The present study aimed to assess the performance and accuracy of ESI in the emergency department of a tertiary care hospital. It will identify the factors that predict under or over triage of patients by the nurses who follow the ESI protocol.

## Materials and methods

A prospective observational study was performed at Hayatabad Medical Complex, Peshawar, from October 2020 to March 2021. The study was carried out after the approval from the ethical committee of Hayatabad Medical Complex was obtained. This study was conducted using data gathered from one of Khyber Pakhtunkhwa's second-largest hospitals.

All patient records for the study period were retrieved since we intended to investigate the demographic dynamics of all patients presenting to our hospital ED. All data from our emergency department was retrieved and recorded using appropriate procedures and software. Patient demographics (age, sex, and nationality), date of visit, acuity allotted by the triage nurse, duration of stay, and resources spent were among the data gathered. A particular intervention is defined as "resources" by the ESI algorithm. Clinical laboratory studies, radiography, electrocardiography monitoring, special studies, fluids, medications intravenous or intramuscular medications, and specialist consultations were all included in the resource-utilization checklist, according to the ESI handbook.

Triage accuracy has been established by comparing proposed resource consumption (acuity level 3-5) to the actual resources utilized in these hospitals as the amount of an agreement between standard guidelines and local observations. In terms of resource expenditure, we also assessed the interconnection between acuity level and extent of accuracy.

All data were analyzed using the Statistical Package for Social Sciences (SPSS version 23, IBM Inc., Armonk, New York) by qualified clinicians once the ESI was implemented. Other statistical conclusions included frequency distributions and correlations. A p-value of < 0.05 was set as statistically significant.

## Results

A total of 9,836 patients presented to the department of emergency at Hayatabad Medical Complex (HMC), Peshawar, Pakistan. The mean age of patients was 38.64 ± 21.42 years. Of those who presented to the ER department during the study, 6067 (61.68%) patients were male. The majority of the patients fell in the triage level three, i.e., 4768 (48.47%) (Table 1). 

**Table 1:** Demographics ESI - Emergency Severity Index, ICU - intensive care unit

Demographics	
Age (mean ± SD)	38.64 ± 21.42
Male	6067 (61.68%)
Female	3769 (38.32%)
Triage level	
ESI 1	41 (0.42%)
ESI 2	1903 (19.35%)
ESI 3	4768 (48.47%)
ESI 4	2967 (30.16%)
ESI 5	157 (1.60%)
Critical outcomes (%)	
ICU admissions	18 (0.18%)
Cardiac catheterization	27 (0.27%)
Surgery	273 (2.78%)
In-hospital mortality	140 (1.42%)

Table 2 illustrates that the odds of under-triage rose with the increasing age of patients. The greatest odds of undertriage to moderate acuity were associated with age ≥65 years; OR 1.49, 95% CI (1.25-1.72) and OR 2.18 CI (1.22-3.73) for under-triage to low acuity designations. Several correlations were noticed between triage vital signs and the likelihood of under-triage when using ESI. Primarily, severe hypoxia (SpO2 ≤89), severe bradycardia, and tachycardia were all strongly linked with risk of under-triage of moderate-acuity levels OR 2.19 95% CI (1.49-3.13); OR 2.54 (1.53-4.01); and OR 2.17 (1.61-2.88), respectively Essentially, there were also significant associations with under-triage to moderate acuity due to the lack of oxygen saturation measurement. Hypertension (≥200mmHg) was linked with increased odds of undertriage to moderate acuity with OR 1.29 CI (0.68-2.01) (Table 2).

**Table 2:** Factors predictive of under-triage to moderate and low Emergency Severity Index acuity levels α (p=<0.05), β (p=<0.01), γ (p=<0.001) ESI - Emergency Severity Index

	Moderate acuity (ESI3), N= 4,768, odds ratio (95%CI)	Low acuity (ESI4 or ESI5), N= 3,124, odds ratio (95%CI)
Age (18–30 years reference)		
30–49 years	1.17 (1.06–1.38)γ	1.09 (1.01–1.17)β
50–65 years	1.42(1.26–1.63)γ	1.51 (1.37–1.65)β
≥65 years	1.49 (1.25–1.72)γ	2.18 (1.22–3.73)α
Sex (male reference)		
Female	0.83 (0.77–0.90)γ	1.02 (0.96–1.07)
Systolic blood pressure (108–176mmHg reference)		
Hypotension (≤99mmHg)	0.85 (0.71–0.99)α	1.06 (0.90–1.24)
Mild hypotension (100–107mmHg)	1.06 (0.87–1.28)	1.14 (1.02–1.27)α
Mild hypertension (177–199mmHg)	1.28 (1.05–1.49)α	1.06 (0.7–1.68)
Hypertension (≥200mmHg)	1.29 (0.68–2.01)	0.82 (0.30–1.91)
Respiratory rate (16-19rpm reference)		
Bradypnea (≤13rpm)	1.61 (0.66–3.32)	0.81 (0.6–1.42)
Mild bradypnea (14–15rpm)	1.21 (1.02–1.43)α	1.11 (0.95–1.23)
Mild tachypnea (20–22rpm)	0.11 (0.83–1.01)α	1.09 (1.01–1.19)
Moderate tachypnea (23–27rpm)	0.86 (0.72–1.10)	1.25 (1.04–1.51)
Severe tachypnea (≥28rpm)	0.98 (0.55–1.61)	1.40 (0.80–2.38)
Temperature (96.3–99.2°F reference)		
Hypothermia (≤94.0°F)	1.14 (0.94–1.38)	0.7(0.71–1.1)
Mild hypothermia (94.1–96.2°F)	0.9 (0.7–1.11)	0.98 (0.84–1.12)
Mild hyperthermia (99.3–100.4°F)	0.92 (0.75–1.09)	1.51 (1.35–1.68)β
Hyperthermia (≥100.5°F)	0.97 (0.76–1.25)	1.66 (1.43–1.91)β
Oxygen saturation (SpO2 >96 reference)		
Severe hypoxia (SpO2 ≤89)	2.19 (1.49–3.13)γ	1.51 (0.86–2.52)
Moderate hypoxia (SpO2 90–94)	1.13 (0.98–1.29)	1.09 (0.93–1.28)
Mild hypoxia (SpO2 95–96)	1.15 (1.04–1.28)α	1.06 (0.96–1.18)
Heart rate (60-104bpm reference)		
Severe bradycardia (≤49bpm)	2.54 (1.53–4.01)γ	0.62 (0.15–1.83)
Mild bradycardia (50–59bpm)	1.21 (0.98–1.49)	1.10 (0.86–1.38)
Mild tachycardia (105–109bpm)	1.07 (0.87–1.31)	1.1 (1.04–1.36)α
Moderate tachycardia (110–119bpm)	1.14 (0.95–1.35)	1.2 (0.99–1.25)
Severe tachycardia (≥130bpm)	2.17 (1.61–2.88)γ	1.46 (1.1–1.93)α

Advanced age, on the contrary, was linked with a lower chance of over triage to high or moderate ESI acuity levels. There were no anomalous vital signs associated with an increased likelihood of over-triage to high and moderate ESI acuity levels (Table 3).

**Table 3:** Factors predictive of over-triage to high and moderate Emergency Severity Index acuity levels α (p=<0.05), β (p=<0.01), γ (p=<0.001) ESI - Emergency Severity Index

	High acuity (ESI1 or 2), N= 1,944, odds ratio (95%CI)	Moderate acuity (ESI3), N= 4,768, odds ratio (95%CI)
Age (18–30 years reference)		
30–49 years	0.78 (0.66-0.91)β	0.81 (0.75-0.85)γ
50–65 years	0.63 (0.53-0.75)γ	0.66 (0.7–0.72)γ
≥65 years	0.39 (0.33-0.48)γ	0.43 (0.38–0.47)γ
Sex (male reference)		
Female	1.39 (1.22–1.58)γ	1.13 (1.07–1.20)γ
Systolic blood pressure (108–176mmHg reference)		
Hypotension (≤99mmHg)	0.72 (0.56-0.92)β	0.86 (0.74–0.98)α
Mild hypotension (100–107mmHg)	1.03 (0.81-1.2)	0.94 (0.85–1.05)
Mild hypertension (177–199mmHg)	0.95 (0.76-1.18)	0.79 (0.66–0.93)β
Hypertension (≥200mmHg)	1.03 (0.76-1.39)	0.99 (0.64–1.49)
Respiratory rate (16-19rpm reference)		
Bradypnea (≤13rpm)	-	1.10 (0.55–2.00)
Mild bradypnea (14–15rpm)	0.78 (0.58-1.06)	1.06 (0.93–1.19)
Mild tachypnea (20–22rpm)	1.06 (0.93-1.20)	1.05 (0.98–1.12)
Moderate tachypnea (23–27rpm)	0.97 (0.80-1.18)	0.88 (0.77–1.01)α
Severe tachypnea (≥28rpm)	0.78 (0.50-1.17)	0.81 (0.50–1.24)
Temperature (96.3–99.2°F reference)		
Hypothermia (≤94.0°F)	0.78 (0.61-1.00)	1.04 (0.96–1.13)
Mild hypothermia (94.1–96.2°F)	1.01 (0.87-1.17)	1.04 (0.90–1.20)
Mild hyperthermia (99.3–100.4°F)	0.99 (0.74-1.34)	0.82 (0.72–0.93)β
Hyperthermia (≥100.5°F)	1.29 (0.90-1.80)	0.67 (0.55–0.80)γ
Oxygen saturation (SpO2 >96 reference)		
Severe hypoxia (SpO2 ≤89)	0.41 (0.26-0.63)γ	0.85 (0.55–1.25)
Moderate hypoxia (SpO2 90–94)	0.73 (0.60-0.89)β	0.61 (0.54–0.69)γ
Mild hypoxia (SpO2 95–96)	0.83 (0.69-0.99)α	0.88 (0.80–0.96)γ
Heart rate (60-104bpm reference)		
Severe bradycardia (≤49bpm)	0.47 (0.21-0.92)α	0.76 (0.40–1.32)
Mild bradycardia (50–59bpm)	1.09(0.81-1.44)	0.99(0.84–1.18)
Mild tachycardia (105–109bpm)	0.84 (0.62-1.12)	0.90 (0.79–1.04)
Moderate tachycardia (110–119bpm)	0.85 (0.67-1.06)	0.98 (0.87–1.10)
High tachycardia (120–129bpm)	0.84 (0.62-1.12)	0.93 (0.77–1.12)
Severe tachycardia (≥130bpm)	0.33 (0.21-0.49)γ	0.87 (0.66–1.15)

## Discussion

The present study assessed the effectiveness of triage by nurses practicing the ESI protocol using clinical judgment with the awareness of adequate resource utilization. The current study revealed that the under-triage of patients was significantly associated with the increasing age of the patient. These findings were similar to a study conducted by Grossman et al., which also noted that the elderly patients were at a much higher risk of under-triage [8]. This implies that older patients are more prone to be a subject of neglect in high-risk cases, associated with the inability to accurately interpret vital signs. The factors that may be associated with the under-triage of such patients might also be linked to communication problems, altered mental status [9], or complexities of the medical and social issues of older patients, which complicates their evaluation [10].

Our study noted that higher acuity levels comprised a greater elderly population as compared to the young. These findings were also found in a similar study by Hinson et al. [11]. This may be associated with the pattern of disease presentation in older adults, which may be linked with the clinically apparent severity of the disease [12]. The relatively higher burden of comorbidity in the elderly population as compared to the young significantly affects the presentation of the disease, making identification of acute illnesses more difficult [13].

Another significant finding in our study was the recording of vital signs comprising heart rate, respiration rate, and saturation of oxygen and its link with the under-triage of the patients. Classifying patients according to Emergency Severity Index protocol requires a proper evaluation of vital signs, as ignorance of irregularities in vital signs, particularly ICU admissions and hospital mortality, are significantly associated with detrimental outcomes [14]. Studies have shown that the correlation of modified vital signs with ESI is positive [15]. Another study compared the vital signs parameters and reported that compared to oxygen saturation, other variables, including the respiratory rate and the heart rate, are more strongly linked to the over-triage of patients from ESI level three to level two [16].

One another finding of the current study was that hypertension (≥ 200mmHg) was linked with increased odds of undertriage to moderate acuity with OR 1.29 CI (0.68-2.01). In contrast to our findings, Hinson revealed that presenting complaints, including hypertension and allergic reactions, were independent predictors of over-triage [11].

Our study was limited due to the small sample size. Research on a larger population must be conducted to better understand the accuracy of ESI in the management of patients requiring acute emergency medical care. More research is warranted, which would focus on the incorporation of several other internal and external factors that can impact the decision-making process of nurses that take part in the ESI assessment protocols. These factors may include years of practitioner experience, education, and hours of training.

## Conclusions

Our study indicated that increasing the age of patients was a significant factor associated with odds of under-triage. Furthermore, certain vital signs, including severe bradycardia, tachycardia, and severe hypoxia, were connected to the risk of under-triage of moderate acuity. Further, large-scale and multicenter studies should be conducted to assess other triage systems, which may provide a more accurate and reliable approach to evaluate the severity of patients’ injuries by the hospital staff and physicians in the emergency room. They should be translated to local languages to assign treatment priorities in a structured and dependable manner.
